# Dealing With Defilement Cases in Ghana: Intricacies of Law, Sociology and Psychology

**DOI:** 10.5964/sotrap.14305

**Published:** 2026-01-21

**Authors:** Jacob Mensah Agboli

**Affiliations:** 1 Narcotics Control Commission, Accra, Ghana; University Medical Center Mainz, Mainz, Germany

**Keywords:** defilement, carnal knowledge, unnatural carnal knowledge, penetration, child sexual abuse, forensic medical examination (FME), Ghana

## Abstract

Defilement is one of the most traumatic experiences a child can experience, with the consequences lived almost throughout the entire lifetime of the victim. Yet, statistics show that defilement cases are on the rise in Ghana, with little being done by the State to stem the tide. This paper critically analyses the incidence of defilement from three perspectives, namely, law, sociology, and psychology. The paper first discusses what defilement is under Ghanaian law (the law), how society deals with the victims and perpetrators of defilement (the sociology), and the psychological effect of defilement on the victims (the psychology). Using a combination of systematic review and the practitioner analysis approach to research, the paper identifies the consequences of defilement on the victim in Ghana, the problems with the definition, scope, and adjudication of defilement cases in Ghana, and offers some useful recommendations to deal with the rising incidence of defilement in Ghana. A major finding of this paper is that the rising cases of defilement in Ghana are the result of the failure of law, sociology, and psychology. Therefore, to combat this canker, an eclectic approach combining the three domains is highly recommended.

## Abstract (plain language summary)

**Background:**

Defilement is a reprehensible conduct in Ghanaian societies and a criminal offence both in Ghana and the world over. Nonetheless, defilement cases have been on the rise, especially in recent times, despite the severe punishment they attract.

**Why was this study done?:**

It has been suggested that the State is not doing enough to fight the menace of defilement. It has further been suggested that the conduct of the Ghanaian society itself contributes to the rising cases of defilement in Ghana. Some have also blamed the victims of defilement as being responsible for their predicament. This paper investigates these suggestions to establish their veracity or otherwise.

**What did the researchers do and find?:**

The paper discusses the offence of defilement under Ghanaian law and critically analyses the sociocultural factors accounting for the continued perpetuation and increase in defilement cases in Ghana. The paper further discusses the psychological effects of defilement on the victims, who are children and adolescents, their families, and society in general. The paper observed that the government has not done enough to deal with the incidence of defilement in Ghana, apart from the criminal sanctions imposed on perpetrators.

**What do these findings mean?:**

For Ghana to address the incidence of defilement and its numerous attendant problems, it is recommended that the State focus more attention on the sociological and psychological aspects of the problem, rather than the legal aspect that currently receives much attention. It is recommended that, for a start, the State pays for the cost of forensic medical examination (FME) and provides psychological treatments for victims of defilement to encourage the victims and their parents/guardians to report defilement cases. This paper offers useful suggestions that, when implemented, will help Ghana significantly reduce the incidence of defilement cases in the country.

A common definition of child sexual abuse (CSA) is any type of maltreatment directed towards a child that involves sexual activity (Hanson & Wallis, 2018). The U.S. Centre for Disease Control and Prevention (CDC) defines CSA as "the involvement of a child (person less than 18 years old) in sexual activity that violates the laws or social taboos of society and that he/she (1) does not fully comprehend, (2) does not consent to or is unable to give informed consent to, or (3) is not developmentally prepared for and cannot give consent to" (CDC, 2022, p. 1). CSA is sexual violence (WHO, 2022) perpetrated against both boys and girls everywhere in the world (Ligiero et al., 2019).

There are several classifications of CSA available (Hanson & Wallis, 2018), such as noncontact sexual abuse (exhibitionism, voyeurism, exposing the child to pornography, and direct physical abuse, with or without penetration (CDC, 2022).

Defilement constitutes a major CSA in the world over, including in Ghana. According to the Ministry of Gender, Children and Social Protection’s (MGCSP) Ghana Action Plan for the Implementation of United Nations (UN) Security Council Resolution 1325 (2000) on Women, Peace, and Security, the media in Ghana have been inundated with stories about defilement that have occurred in schools and communities. In 2017, media attention was drawn to incidents of defilement, notably in the Central Region. The case that received the greatest media attention included the defilement of a 4-year-old girl in Assin Adadientem (MGCSP, 2020).

Globally, up to one billion children between the ages of two and seventeen are thought to be victims of abuse (physical, sexual, or emotional) in 2021 (WHO, 2022). The CDC reports that, in the US, CSA affects roughly 1 in 4 girls and 1 in 13 boys. Of those who experience CSA, 91% are perpetrated by someone the child or the child's family knows and trusts. The lifetime economic cost of CSA in the US was estimated to be at least $9.3 billion in 2015 (CDC, 2022). In Ghana, reporting CSA remains low. Meanwhile, a 2015 study revealed that approximately 27% of females and 11% of boys had experienced sexual assault as children (Owusu-Addo et al., 2023).

Research in Ghana suggests that CSA is high and gendered (Tenkorang, 2023). It has been found that, as gendered-patterned, girls more often than boys are victims of CSA. A study by Quarshie (2021) in Ghana found that 24.3% of teenage girls and 10.4% of teenage boys between the ages of 13-19 and in Senior High Schools in the Greater Accra Region reported having been sexually abused in the immediate 12 months before the survey. Tenkorang (2022) examined sexual violence at sexual debut among Ghanaian youth and discovered that the form of sexual violence was gendered as well; teenage girls reported higher levels of coercion, while boys reported higher levels of forced sex (defilement) at sexual debut.

This paper discusses defilement in Ghana primarily for two reasons. First, defilement is socially abhorred yet prevalent in Ghana. Second, defilement leaves almost permanent scars on the victim, but the State has not implemented comprehensive policy measures to support victims of defilement, whose perpetrators are often close family members, relatives, and friends. Therefore, this paper aims to highlight the challenges of dealing with defilement cases in Ghana and the recommended solutions to deal with the menace of defilement in Ghana.

## Defilement Under Ghanaian Law

Generally speaking, the rape of minors is classified as defilement. Access to justice is crucial for everyone, but it is more crucial for victims of defilement, as defilement is a significant reproductive justice issue (Morhe & Morhe, 2013). Children under the age of 15 make up the majority of rape victims in Ghana (Bortei-Doku & Kuenyehia, 1998; Morhe & Morhe, 2013). According to Bortei-Doku and Kuenyehia (1998), children as young as three months have been defiled in Ghana, and their violators range from as young as fifteen years to elderly men in their eighties.

The offence of defilement in Ghana is codified under section 101 of the Criminal and Other Offences Act, 1960 (Act 29). The said section states as follows:

“Section 101—Defilement of Child Under 16 Years of Age.

1. For purposes of this Act, defilement is the natural or unnatural carnal knowledge of a child under sixteen years of age.
2. A person who naturally or unnaturally carnally knows a child under sixteen years of age, whether with or without the consent of the child, commits a criminal offence and is liable on summary conviction to a term of imprisonment of not less than seven years and not more than twenty-five years.” (p. III-1748).

From the above provision, defilement under Ghanaian law refers to the natural or unnatural carnal knowledge of any child below the age of 16 years. It follows that in Ghana, defilement can occur in one of two ways: either the act is performed without the victim's consent, in a situation that would constitute rape if the victim were sixteen years of age or older, or the act is performed with the victim's consent where the victim is less than 16 years. The discernible philosophy underpinning the definition of defilement in Ghana is that the legislature aims to discourage sexual intercourse, be it natural or unnatural, with children under the age of sixteen years, even if they give their consent to the sexual intercourse.

By the definition of defilement, therefore, the key elements the prosecution has to prove to succeed on a charge of defilement in Ghana are:

1. Natural or unnatural carnal knowledge
2. A child of less than 16 years
3. With or without consent of the child

In the case of *Eric Asante v. The Republic [2017] DLSC 2511*, the Supreme Court of Ghana, speaking through Pwamang JSC, stated as follows:

“The ingredients of the offence of defilement are:

- That the victim is under the age of 16 years (as provided for in Act 554).
- Someone had sexual intercourse with her; and
- That person is the accused.” (p. 2515).

The first element the prosecution has to prove to secure a conviction against an accused person on a charge of defilement is natural or unnatural carnal knowledge. Section 99 of Act 29 explains carnal or unnatural carnal knowledge in the following terms:

“Where, on the trial of a person for a criminal offence punishable under this Act, it is necessary to prove carnal knowledge or unnatural carnal knowledge, the carnal knowledge or unnatural carnal knowledge is complete on proof of the least degree of penetration” (p. III-1748).

In throwing more light on what constitutes carnal knowledge in Ghana, the Supreme Court in the case of *G/CPL Valentino Gligah & EC/1 Abdulai Aziz Atiso v. The Republic [2010] DLSC4149*, stated as follows:

“Carnal knowledge is the penetration of a woman’s vagina by a man’s penis. It does not really matter how deep or however little the penis went into the vagina. So long as there was some penetration beyond what is known as brush work, penetration would be deemed to have occurred and carnal knowledge taken to have been completed” (p. 4153).

It follows, therefore that, regarding carnal knowledge, the prosecution only has to prove the least degree of penetration whenever a male is charged with the offence of defilement of a child. This is the import of the Supreme Court’s decision in the case of *G/CPL Valentino Gligah & EC/1 Abdulai Aziz Atiso v. The Republic [2010] DLSC4149*. Thus, strictly going by the definition of carnal knowledge both by section 99 of Act 29 and the Supreme Court’s decision in *G/CPL Valentino Gligah & EC/1 Abdulai Aziz Atiso v. The Republic [2010] DLSC4149*, carnal knowledge in a defilement case is proved against the accused person whenever the prosecution can establish that the accused person penetrated the vagina of the victim, even by the least degree such as rubbing the tip of his penis against the vulva (labia majora) of the vagina of the victim will be sufficient. Furthermore, by this definition, any form of penetration other than penile penetration will not suffice in proving carnal knowledge.

The second leg of this element is natural carnal knowledge. Thus, unlike the definition of rape that does not recognise other forms of sexual gratification (such as anal or oral sex) than penile penetration of the vagina of a woman (Agboli, 2023), the law in Ghana recognises those other forms of sexual gratification/intercourse in defilement cases. Section 104(2) of Act 29 defines unnatural carnal knowledge as “sexual intercourse with a person in an unnatural manner or with an animal” (p. III-1749). While Act 29 did not state what “unnatural manner” means, it is the submission of the present writer that unnatural manner connotes other forms of sexual gratification/intercourse, such as oral sex or anal sex. This is the import of the Circuit Court’s decision in the case of *The Republic v. Dr. Ali-Gabass*. It is instructive to note, however, that other forms of sexual gratification that are non-penetrative will not satisfy the requirements of unnatural carnal knowledge. This is because section 103 of Act 29 creates the offence of indecent assault, which the law defines as forcibly making a sexual bodily contact with another person without the person’s consent or sexually violating the body of that other person without his/her consent.

In summary, therefore, the element of natural or unnatural carnal knowledge is proved when the prosecution establishes that, the accused person had sexual intercourse with the victim either by penetrating the vagina, anus, or the mouth.

The second element the prosecution has to prove to succeed on a charge of defilement is that the victim is less than 16 years old. It is thus the case that, where the victim is proved to be above 16 years of age, a charge of defilement against the accused will fail. In this case, rape will be the proper charge. The proof of age is an objective one, that is, by resorting to documents such as birth certificates, baptismal certificates, national ID cards, or any other document by which the age of the victim can be ascertained (Agboli, 2023).

The final element the prosecution has to prove in a charge of defilement is whether the sexual intercourse was with the consent of the victim or not. Unlike the case of rape under Ghanaian law, where consent is the most controversial and determining issue/factor (Agboli, 2023), consent is the least controversial issue in adjudicating defilement cases in Ghana. This is because, once carnal knowledge (natural or unnatural) and the age of the victim are established, it is immaterial if the victim consents to the sexual intercourse. In other words, it will be disingenuous for the accused person to claim that the victim gave his/her consent to the sexual intercourse. The accused would still be liable regardless of whether the victim gave his/her consent to being known carnally or unnaturally carnally. For this reason, the crime of defilement is sometimes called "statutory rape", meaning that, the victim's consent does not aid the accused because it is legally void (see section 14(a)). In the case of *Republic v. Yeboah [1968] GLR 248-256*, the High Court held that consent of the victim is no defence in a charge of defilement and that all the prosecution has to prove is that the victim was carnally or unnaturally carnally known by the accused person. In this case (*Republic v. Yeboah*), the court found that the victim, who was just 9 years old, was carnally known by the accused.

In the case of *Republic v. Dr. Sulley Ali-Gabass*, a medical doctor at one of the country’s hospitals tied up a boy who was less than 16 years of age at the time of the defilement and forced the victim to have anal sex with him (Dr. Ali-Gabass) in his (the perpetrator’s) vehicle. Afterward, the victim got infected with HIV. Investigations revealed that the perpetrator took advantage of the victim’s poor background and bought the victim gifts like a Samsung Galaxy phone and gave him 20 Ghana cedis, which was about the equivalent of 5 dollars at the time. Although the perpetrator denied any wrongdoing, an investigative journalist in an undercover interview managed to obtain a confession from Dr. Ali-Gabass on camera. Consequently, Dr. Ali-Gabass was arrested, charged, and prosecuted on charges of defilement. After a long trial, Dr. Ali-Gabass was convicted and sentenced to 25 years imprisonment in hard labour. The presiding judge, Mrs. Rita Agyeman-Budu, in convicting Dr. Ali-Gabass stated as follows:

“There is no doubt in my mind that the accused person had anal sex with the victim who, at the time of the act, was under 16 years and I find the accused guilty on the charge of defilement, contrary to Section 101(2) of Act 29, 1960 and convict him accordingly. He is hereby sentenced to 25 years imprisonment, which is the maximum sentence for defilement in Ghana… It will serve as a deterrent to people who subject minors, whether male or female, to such sexual acts amounting to violence. This act of the accused is very reprehensible and I condemn it in no uncertain terms” (p. 12).

Mrs. Agyeman-Budu, quoting the renowned Supreme Court Judge, Justice Jones V.M. Dotse, further stated that,

“There is no doubt that as a nation, apart from narcotics, which is a menace, defilement cases are on the increase. It, therefore, leaves no one in doubt that there is the need for a concerted effort to remove and destroy this dangerous canker of defilement from our society…The moral decadence into which the country has sunk makes it imperative for all and sundry, especially the law enforcement agencies like the courts, to be at the vanguard of this crusade” (p. 14).

## Proof in Defilement Cases in Ghana

The general rule of law is that, a person who alleges must prove his/her allegations. Thus, the person who makes an allegation of wrongdoing against another carries the burden of proving his/her allegation against that other person. It is only when the person making the allegations discharges his/her burden of proof that the burden shifts to the defendant/defence to rebut the proof. Accordingly, section 11(2) of the Evidence Act of Ghana, 1975 (NRCD 323) provides that, in criminal matters, the prosecution carries the burden of proving its case/allegation against the accused beyond reasonable doubt. It follows, therefore, that in defilement cases, the prosecution carries the burden of proving its allegation against the accused person (perpetrator) beyond reasonable doubt. In other words, the complainant/prosecution must establish in a defilement case that; (1) the victim was carnally or unnaturally carnally known; (2) that the person who carnally or unnaturally carnally knew the victim is the accused person; and that (3) the victim was below the age of 16 years. Typically, prosecution of defilement cases commences by the victim, a relative, or any person who becomes aware of the victim's ordeal making a complaint to the police about the incident. Since in all cases, the victims are children, sometimes infants/toddlers/babies, the parents, relatives, or guardians often make the complaint on their behalf. It is also the case that, in many cases, the victims might have been threatened with violence by their violators if they report, enticed with gifts not to report (as in the case of Dr. Ali-Gabass), or voluntarily yield to the demand of the perpetrators (as in the case of *Republic v. Yeboah [1968] GLR 248-256*). Thus, the victims do not report the case at all or reluctantly do so some days after the ordeal. In Ghana, unreported CSA sometimes prevent the victim from receiving a variety of interventions, including psychological, medical, and legal assistance.

In some cases, the victims’ parents/guardians notice changes in their physique/stature, posture, or even witness blood oozing out of their private part(s) which had been penetrated to become aware that their child has been defiled and complain to the police. Thereafter, the victim is issued with a police medical form to visit any medical facility (public or private) to be examined by a licensed medical practitioner.

To prove carnal or unnatural carnal knowledge, the prosecution must lead evidence to establish that, either the accused person [voluntarily] confessed to carnally or unnaturally carnally knowing the victim, or that the prosecution has gathered enough incriminating evidence in proof of the accused person’s guilt as far as proof of carnal or unnatural knowledge is concerned. According to Agboli (2023), to prove carnal or unnatural carnal knowledge in cases of sexual assault (in this case defilement), the police use forensic medical examination reports—that is, a medical report on a police medical form (CID From 98A) signed by a licenced medical professional who examines the victim, testifies to the report, and is available for cross-examination on the said report. According to Berger et al. (2023), “forensic medical and gaenecological examination after sexual assault is an essential procedure, yet it is a potentially further traumatizing experience for the victim” (p. 848). Typically, the forensic medical examination (FME) comprises obtaining a patient's medical history, performing a comprehensive physical and genito-anal examination and documenting any injuries found, gathering forensic specimens, and providing medical care, including managing STIs and pregnancy (Berger et al., 2023; Cybulska, 2013; WHO, 2003). But other aspects of the examination—like the use of colposcopy or toluidine blue staining—require following local protocols (Berger et al., 2023; Ingemann-Hansen & Charles, 2013).

Age is the second and last element to prove in a defilement case. To prove this, the prosecution/police would have to provide the victim's birth certificate or any other document that may be used to determine the victim's age. A passport, national identity card, national health insurance card, and baptismal certificate are a few examples of these documents. Any document that might be used to determine the victim's age and date of birth would be sufficient in this regard (Agboli, 2023). In the case of *Republic v. Yeboah [1968] GLR 248-256,* for example, the baptismal certificate of the victim was used to prove her age.

## Competence of Defilement Victims to Testify in Court

The victims of defilement are always children, some as young as 19 months (Ghana News Agency, 2023) or 2 years old (Fatawu, 2020). Given that the victims of defilement are children, a thorny yet less-examined issue in the adjudication of defilement cases is the competence of defiled children to testify in court.

Historically, the courts have been circumspect about the testimony of child witnesses. Two reasons underpinned this circumspection. First, child witnesses were thought to be inherently unreliable, suggestible, and prone to false allegations, particularly in cases of sexual abuse. Second, child witnesses were believed to be unable to demonstrate an understanding of the nature and consequences of an oath. As such, for years, competence inquiries acted as an initial, critical obstacle, preventing children from giving evidence in court (Brubacher et al., 2019).

Under section 59 of the Evidence Act of Ghana, 1975 (NRCD 323), a child is competent to testify in court so far as the child is capable of expressing himself/herself to be understood directly or through an interpreter or is capable of understanding the duty of a witness to tell the truth (Brobbey, 2014). It follows, therefore, that evidence from a child witness before the court is admissible once these conditions set in section 59 of NRCD 323 are met and there is no need for corroboration of such evidence/testimony from the child, as provided for by section 7(3) of NRCD 323. Similar standards exist in many common law jurisdictions as well. For example, in the US, the US Supreme Court made the first ruling allowing children to testify in court in 1895, accepting a 5.5-year-old as a witness (Pantell, 2017). In the United Kingdom (UK), the Youth Justice and Criminal Evidence Act 1999 (YJCEA 1999) states in section 53(1) that "At every stage in criminal proceedings, all persons (whatever their age) are competent to give evidence" unless the court is of the view that the witness is incapable of understanding questions posed to him or her and providing comprehensible answers (Crown Prosecution Service, 2018). Similar standards exist in Canada (Westman, 2018), Australia (Brubacher et al., 2019), Nigeria (Smaranda & Babalola, 2019), and Kenya (Nzisa, 2020).

According to III (1953), there was thought to be a cutoff age legally, somewhere between 9 and 10 years old, below which no child could serve as a witness. Unfortunately, as the courts found themselves powerless to punish crimes committed against or in the presence of children, the unfavourable effects of this legal thought/imposition grew more and more apparent. At the same time, the legislature also did not seem to demonstrate its willingness to take action to address the issue. As a result, in the seminal case of *Rex v. Brasier (1779) 1 Leach 199*, the courts took the lead in shaping the contemporary legal framework regarding the competency of children to testify in court. The defendant in this case was accused of attempting to rape a child who was younger than seven years old. The child victim was deemed incompetent to testify, but the defendant was found guilty based on the hearsay testimony of the child's mother and a friend. Following an appeal, the court ruled in favour of the defendant but held that a child who is old enough to understand the significance of an oath and its obligations is competent to testify in a criminal trial.

Thus, since the case of *Rex. v. Brasier*, the principle now is that a child is competent to testify in criminal cases, either as a victim or an eyewitness, provided the child understands the oath to tell the truth and is capable of expressing himself/herself in comprehensible language to be understood by the court.

## Consequences of Defilement on the Child/Victim

According to the CDC, a serious public health issue and adverse childhood experience (ACE) is CSA (CDC, 2022). Sexual abuse is among the most traumatic experiences a child can go through in his/her lifetime (Berger et al., 2023). It has major short and long term effects on mental and physical health, including the possibility of physical harm, unintended pregnancies, STIs, and a higher risk of depression, anxiety, chronic pain, drug and alcohol abuse, and even suicide (Gilmore et al., 2020; Zinzow et al., 2012). CSA has also been linked to personality disorders and conversion disorders (Hailes et al., 2019). As reported by Mutavi et al. (2016), defilement is a traumatic experience that is frequently linked to psychological issues in children, parental anxiety, and major social pressures on happy, healthy families. In a study conducted between June 2015 and July 2016 at Kenyatta National Hospital and Nairobi Women's Hospital in Kenya, Mutavi et al. (2016) examined the psychosocial outcomes in defiled children and their caregivers' perceptions of the children's trauma after defilement. They found that the defiled children had negative outcomes such as poor academic performance, low self-esteem, depression, and poor social relationships. Furthermore, two of the children were pregnant, one of them used drugs, another had a mental disability, and one of them contracted HIV/AIDS.

Similarly, according to the American College of Obstetricians and Gynaecologists (2017), the most often reported emotional effects of sexual abuse in childhood are depression, anxiety, and anger. Among survivors, gynaecological issues are frequently diagnosed, including nonspecific vaginitis, dyspareunia, persistent pelvic pain, vaginismus, and gastrointestinal disorders. Those who have survived may be less likely to get regular Pap tests and may not seek out much or any prenatal care. It has further been found that disturbances of desire, arousal, and orgasm may result from the association between childhood sexual activity, violation, and pain. Survivors are more likely to have had 50 or more intercourse partners, have had a sexually transmitted infection, and engage in risk-taking behaviours that place them at risk of contracting HIV. Early adolescent or unintended pregnancy and prostitution are associated with sexual abuse (American College of Obstetricians and Gynaecologists, 2017). It has been estimated that between 30-53% of HIV-positive infections are due to defilement (Lo Iacono et al., 2021; McCall et al., 2018), the infections being largely attributable to behavioural and psychosocial factors, such as engaging in risky sexual behaviours. While little research has been done in these areas in Ghana and reported in the literature, the present writer believes that these findings reflect the experiences of defilement victims in Ghana.

The study by Mutavi et al. (2016) further found that not only is defilement traumatic to the child victim, but it also negatively affects the parents and caregivers of these children. As such, significant psychosocial distress was experienced and reported by the caregivers of children who suffer defilement. This situation observed in Kenya is not different from those experienced by child defilement victims in Ghana. In Ghana, Morhe and Morhe (2013) found that, long after the act has been committed, defilement victims continue to experience negative psychological and financial repercussions.

The FME itself can be extremely traumatic for the victim, who is already *suffering* over the defilement incident. As a result, the victim, who is a child, experiences double agony of trauma. According to Berger et al. (2023), to prevent further traumatisation of the victim, it is essential to guarantee ideal examination settings that also address emotional demands, in addition to the medico-legal standards. Out of 49 sexually assaulted women surveyed by Berger et al. (2023), 52% of the participants perceived the FME as an additional psychological burden to their already traumatising experience. This is not surprising because, in the course of the FME, the victim must recall the event, report it, and end up reliving the event again.

Another common consequence of defilement on the victim is pregnancy. In several cases, female victims of defilement become pregnant, presenting a double agony for them. Some of these victims, who have had to succumb to the sexual demands of men in exchange for favours, gifts, and money, sometimes become pregnant and are, in some cases, abandoned by their violators. It must be emphasised that, since consent is immaterial in defilement, even if the victim consents to sexual intercourse with the perpetrator in exchange for some financial or material gains, that consent is inoperative in exculpating the perpetrator from liability. When they become pregnant as a result of the defilement, the pregnancy not only reminds the victims of their ordeal, but the children born also become burdens on their child mothers. When the perpetrator is prosecuted and imprisoned, the worry for these child mothers is worsened, as they have to fend for themselves and their children alone. In consequence, according to Morhe's assessment of the Ewe of Ghana's informal courts, most families of defilement victims prefer not to pursue legal action when the offence results in pregnancy because the victim and the unborn child's welfare come first (Morhe, 2011; Morhe & Morhe, 2013). Thus, offers of money are made and frequently accepted in place of legal action (Morhe, 2011; Morhe & Morhe, 2013). Meanwhile, defilement in Ghana is a first degree felony that cannot be settled amicably or by resort to alternative dispute resolution methods.

Still on the issue of pregnancy, Morhe and Morhe (2013) observed that defilement victims in Ghana also become vulnerable to STDs like HIV/AIDS as well. A large number of them also develop susceptibility to long-term health issues linked to early motherhood. According to statistics from the DOVVSU, 1,047 incidents of defilement were among the 2,865 pregnancy cases in girls aged 10 to 14 that were reported to the DOVVSU in 2020 (GNA, 2022).

There is no gainsaying that the pregnancy of girls between the ages of 10 and 14 is the result of defilement. Yet, there is a significant discrepancy between the number of documented cases of teenage pregnancies among girls under the age of 16 and the reported cases of defilement. This suggests that the majority of the defilement cases in Ghana are unreported. Secondly, strictly speaking, the men who got all these girls pregnant should have been prosecuted, since they had sex with children (girls) below the age of 16 years, with or without their consent. Yet, social pressure and poverty compel these girls not only to refuse to report their ordeal to the police for their violators to be prosecuted (Agboli, 2023) but also they fear becoming single mothers, having to take care of their children alone. The consequence is that parents and/or guardians frequently accompany defilement victims to file complaints of neglect and/or denial of pregnancy *against the perpetrators at the Social Welfare Department* when investigations reveal that the girls were impregnated through sexual abuse, instead of reporting the offenders to the police for prosecution. As found by research, 40% of the instances of teenage pregnancy had direct reports of sexual abuse, whereas 60% of the cases were indirect reports (Agordzo Edoh-Torgah & Matafwala, 2022).

## Problems With the Definition, Scope, and Adjudication of Defilement in Ghana

Ghana inherited its legal system largely from its colonial masters. The law on defilement, therefore, is one of those laws inherited from Britain and continued under Act 29 when Ghana gained Republican status in 1960. As good as the law prohibiting defilement is, to the extent that it protects children from sexual violations and exploitation, it is also fraught with several issues/problems that need to be addressed to bring Ghana’s law in line with those of other common law countries.

One major issue/problem with the definition, scope, and adjudication of defilement cases in Ghana is the age of consent to sex. As already noted, sexual intercourse with any person below the age of 16 years, whether consensual or not, amounts to defilement under Ghanaian law. By this provision, the age of consent to lawful sexual intercourse in Ghana is set at age 16. Meanwhile, the age of consent to marriage in Ghana is 18 years. Therefore, while a person can have legal sexual intercourse with another at the age of 16, s/he cannot marry and have sexual intercourse with the spouse until s/he attains the age of 18. Commenting on the challenges posed by these legal complexities, the United Nations Children’s Emergency Fund (UNICEF) noted that, to guarantee proper protection for children, other state institutions—like the Ghana Police Department's Domestic Violence and Victims Support Unit (DOVVSU) and the Ministry of Gender, Children and Social Protection (MCGSP)—have been advised to make child sex a crime (UNICEF Ghana, 2008). However, attitudes on the legal age of sexual consent have also been influenced by sociocultural norms. There is no recognised and accepted age of consent in the majority of Ghanaian social, cultural, and religious contexts (UNICEF, 2019). It is to be noted that, before marriage, sexual activity is typically forbidden by Ghanaian customs and values (Gyekye, 2003; UNICEF, 2019). In consequence, having sex is normally acceptable as long as a person is married, regardless of age. It is important to highlight that there have not been many legal interventions, despite the concerns raised about the difference in the legal age of sexual consent and marriage. The effort to prevent child marriages has received the most attention thus far.

As noted by UNICEF (2019), it appears that not much attention has been paid to Ghana's legal age of sexual consent. Meanwhile, there are significant ramifications for Ghana if the current norm surrounding the age of sexual consent is altered or maintained. It means that more young teenagers would be found guilty of major sexual offences like defilement, which have grave repercussions, if the age of sexual consent were raised to equal the age of consent to marriage (18 years). As a result, boys and girls who consent to intercourse at the age of 17 will be considered defiled. This result would seem excessively severe, given that the legislation against defilement primarily targets adults who sexually violate children, not young people who are only having sex experiments (Thomson, 2004; UNICEF, 2019). On the other hand, parents and educators may be able to teach children about sex and related activities if they choose to keep the age of sexual consent at 16 or lower it, while keeping the age of consent to marriage at 18 (Oberman, 2000; UNICEF, 2019).

It follows that the Ghana legal regime, as it currently exists, presents an irony of controversies. This is because, by law, a child can consent to sex once s/he attains age 16. However, that same child cannot consent to marriage in Ghana (de Groot et al., 2018), a social institution in which sexual intercourse is a key feature and procreation is highly valued. At the same time, culturally, there is no age limit for marriage, and the state has had to contend with many cases of child marriages (UNICEF, 2019). As observed by UNICEF, in Ghana, 1 in 5 girls between the ages of 20 and 24 gets married before turning 18 (UNICEF, n.d.). It has further been observed that, since premarital sex and childbirth bring shame to the family, child marriage is used as a safeguard against promiscuity (Ahonsi et al., 2019; Malhotra, 2010). Early marriage is encouraged since it is socially unacceptable in traditional Ghanaian societies for children to engage in premarital sex and childbearing (Ahonsi et al., 2019). The result is that, contrary to the provisions of the law on defilement, many children (in this case, young girls) are being given in marriage by their parents and bearing children, an act which amounts to both the criminal offence of child marriage (a misdemeanour) by the parents under section 109 of Act 29 and defilement by their husbands under section 101 of Act 29. Nonetheless, we have a situation where the law is behind society, such that the practice of child marriage and its attendant defilement of the girl child is being perpetrated almost daily in Ghanaian communities (Ahonsi et al., 2019).

Additionally, a serious challenge with the adjudication of defilement cases in Ghana is the manner such cases are conducted. In most cases, once the victim is capable of speaking for himself/herself and understands the meaning of the oath to tell the truth, the victim is the principal witness for the prosecution. As a result, the victim is made to recall and narrate the incident/ordeal in court to the hearing of all present. Before this, the victim would be met and prepared (coached) by the state prosecutor on how to testify in court. Unfortunately, however, the focus is on the victim’s ability to recall the event vividly to the court, to paint the real picture for the judge to, perhaps, experience the same emotions the victim experienced. However, there is little to no attention paid to the trauma the victim experienced during and after the ordeal. The result is that the victim is made to relive the traumatic experience again, this time in the courtroom, with no psychological intervention to psyche him/her up, first, to face the perpetrator in court and second, to recall the ordeal without breaking down. In the case of *Republic v. Yeboah,* for instance, the victim was a 9-year-old girl who was in primary 1 at the time of being defiled by the accused person, who lives in the same house with the victim. The child victim narrated her ordeal as follows:

“I was wearing a cover cloth with pants underneath it. The pair of pants I had on had elastic fitted at the top of them. Before the accused put me on his thighs, he took off my cover cloth and also my drawers or pants. He put me on his thighs with my face towards him. My legs were hanging and could not touch the ground. My legs were apart with one on the left side of the accused and the other on the right side of the accused in this fashion I am going to demonstrate to the court as I sit on the thighs of this woman seated in front of the court. [By court: Witness demonstrates the position.] The accused then put his penis inside or into my vagina, and I cried as I was feeling pains. He pushed his penis into my vagina. As he kept on doing that I saw some whitish liquid coloured a bit with red in my vagina. The liquid was both white and red i.e. it was a mixture of the two colours. It came from my vagina. It was when I got up that I saw that the said whitish-reddish liquid coming from my vagina. I had before that incident never seen that whitish-reddish liquid ever before in my vagina. When I got up and saw that liquid in my vagina, I did not do anything. I just put on my pants and as I was going home the accused gave me a two-and-a-half new pesewas piece saying I should buy some food with it. I also put on my cover cloth before going away from the accused's workshop where the accused had sexual intercourse with me” (p. 250).

One can imagine a 9-year-old child having to narrate this ordeal a year after experiencing it. However, there is no record to show that, before this narration in the courtroom, in the full glare of the perpetrator, the lawyers, the judge, and all present, the child was psychologically prepared to face her violator (who lived in the same house with her and who she is quite acquainted with) and the court. There is equally no evidence that after reliving the ordeal by having to narrate the experience in court, she was offered any psychological assistance to deal with the PTSD and other psychological consequences she might develop in later years. Meanwhile, several acute and long-term psychological and physical health issues, such as depression, PTSD, substance abuse issues, and sexual victimisation during adolescence and adulthood, have been linked to CSA (Cutajar et al., 2010; Dube et al., 2005; Hanson & Wallis, 2018). Additionally, Guerra et al. (2018) found from their study on 106 female adolescent victims of CSA that self-efficacy was inversely correlated with symptomatology and positively correlated with active coping, as well as negatively correlated with violent sexual abuse, as in this case.

Furthermore, research has shown that children become nervous before court appearances, and their primary concern is looking up at the offender. Additional concerns include being harmed by the accused, feeling ashamed of sobbing or being unable to respond to inquiries, and ending up in jail (Pantell, 2017). Children are less able to respond to questions the more terrified they are. The degree of abuse and the age of the victim are the biggest indicators of insufficient responses (Pantell, 2017). Emotional challenges arise from postponements, and repeat testimony is linked to long-term mental health issues. Shielding processes, including using a two-way video monitoring system to testify, are less stressful for children than making court appearances, and children who testify under shield give more precise and in-depth answers. Yet, Ghana has no policies in place to address these issues reported in the literature.

Another problem with the adjudication of defilement cases in Ghana is the duration of the trial. As a first degree felony in Ghana, the law requires that defilement cases must be tried by a judge and jury, with the numerous challenges associated with jury trials leading to its abolishment (or total reformation) in some jurisdictions (Agboli, 2023). On average, trials of defilement cases take not less than 6 months to a year. This is in cases where there are fewer adjournments and all stakeholders (the police, the complainant, the court, etc.) demonstrate their willingness and commitment to prosecute the case expeditiously. According to Pantell (2017), the general anxiety experienced by children when they have to testify in court is aggravated by delays in the prosecution of the case. In the UK, victims-survivors of sexual abuse who had to wait two to three years for a resolution of their cases described themselves as "living in limbo" and having "no road map" for how to proceed with the criminal justice system or with their lives in general, particularly when there was a dearth of communication about what was going on and why (Burman & Brooks-Hay, 2020). Similar experiences exist in Ghana for victims of defilement.

Apart from the above, a major problem with the adjudication of defilement cases in Ghana is the lack of psychological support/treatment for the victim. It is the case that, not only do children who suffer defilement receive no psychological support in preparing them to testify in court and face the people who have offended against them but also that, they do not receive any psychological treatment/support for the ordeal they have experienced after testifying in court and/or when the trial is over. According to Hanson and Wallis (2018), referrals to professionals trained in the delivery of evidence-based trauma-focused interventions are essential for individuals experiencing serious mental health issues associated with CSA, even though not all victims will show symptoms that call for mental health treatment services.

Also, just like the victim, the perpetrator receives no (psychological) treatment for his sex offending. Meanwhile, research has shown that not only is treatment/psychological intervention necessary for those who perpetrate sexual offences against children but it also reduces recidivism (Sousa et al., 2023). Unfortunately, at the moment, there is no intervention, treatment/rehabilitation services for perpetrators of defilement in Ghana. They neither receive any psychological assessment nor medical treatment (Agboli, 2023). They are simply tried, convicted, and imprisoned, where they spend between seven to 25 years in hard labour without going through any form of treatment.

Furthermore, a challenge with the adjudication of defilement cases in Ghana is poverty. Because of the poor status of many victims of defilement, they are unable to pay for the cost of FME, coupled with the cost of psychological treatment for the victim. This meant that either the victim decides not to report the case at all, or after reporting, is compelled to withdraw from the case (Agboli, 2023). Meanwhile, the FME report is the only acceptable way to prove carnal or unnatural carnal knowledge in Ghana (Agboli, 2023).

## Conclusion and Recommendations

One of the recommendations of this paper is the reform of the law relating to testimony of child witnesses in defilement cases. The current position where children testify on oath and swear to tell the truth appears quite outmoded. The two conditions precedent to the competence of child witnesses, to wit, swearing an oath to tell the truth, and understanding what the oath to the tell the truth means, pose serious challenges for such child witnesses and the credibility of their testimonies. In this regard, it is suggested that, instead of swearing an oath, child witnesses should merely make a promise to tell the truth. As noted by Brubacher et al. (2019), reforms aimed at removing the barrier caused by competency standards have been implemented in recent years. In Australia for example, the 2017 Criminal Justice Report of the Royal Commission into Institutional Child Sexual Abuse (‘Royal Commission’) 85 recommendations (Recommendation 62 concerned children’s competence to give evidence in court) revolutionalised the law on the competence of children to testify, especially in CSAs (Brubacher et al., 2019). The Royal Commission recommended using straightforward, non-theoretical questions to determine a child witness's competence and requiring witnesses who lacked the competence to give sworn testimony to "promise" to tell the truth and then be made to give unsworn testimony. Children now have more opportunities to testify when they are unable to meet the competency threshold for sworn testimony, thanks in part to the introduction of unsworn evidence. Thus, instead of giving a sworn testimony on oath, a child witness could be made to give an unsworn testimony.

In the US, structural and policy changes have been introduced to aid child victims of sexual abuse in testifying in court. According to Pantell (2017), various accommodations were established to lessen the stress that children faced when testifying in court. These included allowing children to hold familiar objects and having a support person accompany them during the testimony process. It has recently been permitted for facility dogs with specialised training to provide consolation to witnesses (www.courthousedogs.com).

It is further recommended that child victims of defilement in Ghana should be given psychological treatment for their experience. Studies have indicated that most trauma-focused interventions with empirical support for children and teenagers are cognitive behavioural therapies (CBTs), which have several common components. Affective modulation techniques like breathing exercises and relaxation techniques, cognitive processing to correct false or harmful beliefs like guilt or self-blame, and progressive exposure to trauma memories are some of these components. Psychoeducation regarding trauma and its effects (e.g., PTSD) is another. The body of research demonstrating gradual exposure's unique ability to lessen PTSD symptoms suggests that it is a particularly crucial component of treatment (Deblinger et al., 2011; Dorsey et al., 2011; Hanson & Wallis, 2018; Nixon et al., 2012; Salloum & Overstreet, 2012). To put it briefly, this entails repeatedly being exposed to the specifics of the trauma to eradicate any emotional or behavioural effects associated with it. It has been shown that using this therapeutic approach to enhance cognitive processing of the traumatic event(s) aids in healing. Participation of a supportive carer in trauma-focused treatments can be another significant factor related to positive outcomes, such as decreased dropout rates (Chowdhury & Pancha, 2011; Hanson & Wallis, 2018), higher family engagement, and enhanced parent-child relationships (Hanson & Wallis, 2018; Ippen et al., 2011). This is evident with most child mental health treatment interventions. This can be achieved by the state engaging the services of clinical psychologists and paying for their services using the National Health Insurance. In cases where the perpetrator has the means, s/he must be made to pay for the treatment of the victim.

Furthermore, the perpetrators must equally be treated. Merely imprisoning them is not enough to end their sex offending proclivities. Rather, such persons have been proven to benefit immensely from psychological treatment techniques such as relapse prevention (RP), risk-need-responsivity (RNR) principles, good lives models (GLM), and Young’s schema-focused models (Sousa et al., 2023). These techniques have been found not only to treat sex offending but also to prevent recidivism. For example, within the domain of sexual offending, Young's schema-focused models facilitate an enhanced understanding of the cognitive and developmental mechanisms involved in sexual offending, to develop coping strategies to overcome them. This can be achieved by recruiting trained and clinical psychologists with experience in treating sex offenders into the Ghana Prison Service, whose duty would be to treat convicts of defilement. The state can also engage clinical psychologists in private practice for this purpose. Considering the fact that Ghanaian society is largely characterised by mechanical solidarity, any successful approach in this regard must be multidimensional, involving the victim, families, and sometimes religious groups.

Finally, the state must institute policy measures to confront the defilement menace head-on. For example, the state must put in place policies that take care of children conceived as a result of defilement, if the mother and her family are unable to do so. In this regard, the Social Welfare Department of the MGCSP must be strengthened and resourced to take care of both the mother and child, in the absence of the perpetrator-father due to imprisonment. This will go a long way to encourage victims of defilement to report the offence and prosecute the case to its logical conclusion.

Moreover, the State must pay for the cost of the FME for defilement victims. This can be catered for either under the DOVVSU Fund or the National Health Insurance Scheme. This will go a long way to encourage victims of defilement to report their ordeal and have their cases successfully adjudicated.

Also, defilement victims must be compensated by their perpetrators when the perpetrators are adults. Where the perpetrators are children or adults of no means, the State must take up that responsibility. This gives the victim some form of reparation and also serves as a deterrent for the perpetrator and like minds. The lack of compensation for victims is one factor deterring victims from reporting defilement cases in some situations. It will also prevent the settlement of defilement cases (Agboli, 2023) where victims receive pittances (Akonor & Okorley, 2021) to withdraw their cases from the police.

In addition, the MGCSP must intensify public education on defilement in Ghana. For example, under section 1 of the Alternative Dispute Resolution Act, 2010 (Act 798), defilement cases cannot be settled by ADR. Yet, defilement cases are being settled almost daily in our communities. Even after reporting the case, the victims and/or their families are pressured to withdraw the case or settle for a pittance in terms of compensation. In some cases, the child victims are married off to the perpetrator. Public education will stem this tide.

Finally, what is clear from this paper is the fact that defilement in Ghana is the result of the intricacies of law, sociology, and psychology. Thus, dealing with the crime will also require an eclectic approach that tackles the canker from three perspectives. It is thus recommended that the State takes steps not only to deal with defilement legally but also sociologically and, most importantly, psychologically by discouraging families of victims from settling cases or pressuring the victims to do so, and providing psychological treatment for victims and perpetrators. So far, Ghana’s approaches to dealing with defilement have been largely legal, and this accounts for the failure of the approach and the increasing number of defilement cases in the country.

## Social Media Accounts

*Jacob Mensah Agboli:* Twitter (X)
